# Assessment of lesion-associated myocardial ischemia based on fusion coronary CT imaging – the FUSE-HEART study: A protocol for non-randomized clinical trial

**DOI:** 10.1097/MD.0000000000025378

**Published:** 2021-04-09

**Authors:** Alexandra Gorea Stanescu, Imre Benedek, Diana Opincariu, Roxana Hodas, Mihaela Ratiu, Theodora Benedek

**Affiliations:** a Department of Cardiology, University of Medicine, Pharmacy, Sciences and Technology of Targu Mures; b Department of Advanced Research in Multimodality Cardiovascular Imaging, Cardio Med Medical Center, Targu Mures; c Department of Radiology, University of Medicine, Pharmacy, Sciences and Technology of Targu Mures, Romania.

**Keywords:** coronary computed tomography angiography, fusion imaging, inflammation status, myocardial ischemia, myocardial viability, plaque composition, plaque vulnerability

## Abstract

**Introduction:**

Multimodality assessment of coronary artery lesions has demonstrated superior effectiveness compared to the conventional approach, for assessing both anatomical and functional significance of a coronary stenosis. Multiple imaging modalities can be integrated into a fusion imaging tool to better assess myocardial ischemia.

**Material and methods:**

The FUSE-HEART trial is a single center, prospective, cohort study that will assess the impact of a coronary artery stenosis on myocardial function and viability, based on advanced fusion imaging technics derived from Cardiac Computed Tomography Angiography (CCTA). Moreover, the study will investigate the correlation between morphology and composition of the coronary plaques and myocardial ischemia in the territory irrigated by the same coronary artery. At the same time, imaging parameters will be correlated with inflammatory status of the subjects. The trial will include 100 subjects with coronary lesions found on CCTA examination. The study population will be divided into 2 groups: first group will consist of subjects with anatomically significant coronary lesions on native coronary arteries and the second one will include subjects surviving an acute myocardial infarction. The vulnerability score of the subjects will be calculated based on presence of CCTA vulnerability markers of the coronary plaques: napkin ring sign, positive remodeling, spotty calcifications, necrotic core, and low-density plaques. 3D fusion images of the coronary tree will be generated, integrating the images reflecting wall motion with the ones of coronary circulation. The fusion models will establish the correspondence between plaque composition and wall motion in the subtended myocardium of the coronary artery. The study primary outcome will be represented by the rate of major adverse cardiac events related to myocardial ischemia at 1-year post assessment, in correlation with the degree of coronary artery stenosis and myocardial ischemia or viability.

The secondary outcomes are represented by the rate of re-hospitalization, rate of survival and rate of major adverse cardiovascular events (including cardiovascular death or stroke), in correlation with the morphology and composition of atheromatous plaques located in a coronary artery, and myocardial ischemia in the territory irrigated by the same coronary artery.

**Conclusion:**

In conclusion, FUSE-HEART will be a study based on modern imaging tools that will investigate the impact of a coronary artery stenosis on myocardial function and viability, using advanced fusion imaging technics derived from CCTA, sighting to validate plaque composition and morphology, together with inflammatory biomarkers, as predictors to myocardial viability.

## 1 Introduction

### 1.1 Background and rationale

Multimodality appraisal of coronary artery lesions in patients surviving an acute myocardial infarction (AMI) has demonstrated superior effectiveness in the complex evaluation of coronary circulation and residual lesions after percutaneous coronary interventions (PCI). Having a specificity of 85% for the diagnosis of coronary artery disease, Cardiac Computed Tomography Angiography (CCTA) offers also the possibility to evaluate the morphology and structure of atheromatous coronary plaques, at the same time providing a detailed view on the cardiac geometry using 3D reconstruction of acquired images.^[1–3]^

Coronary plaques were intensively studied using CCTA-based software for investigation of plaque morphology, which identified specific characteristics of vulnerable plaques that makes them prone to rupture. Usually, these vulnerable coronary plaques contain a necrotic core, spotty calcifications, have positive remodeling and a thin cap fibroatheroma.^[4–7]^

Different scores have been proposed for quantifying the impact of a coronary artery stenosis on the myocardial function. For instance, Duke Jeopardy score is a relatively simply scoring system that expresses the percent of the affected myocardium.^[8]^ The main reason for investigating the impact of a coronary stenosis on the myocardial contraction after myocardial infarction is to identify the presence of viable myocardium, which may restore its functionality and contraction after a proper revascularization procedure.^[9]^ However, in an animal experiment, Force et al. found that viable myocardium juxtaposed to widespread scarring may be unable to recover after proper revascularization due to tethering.^[10]^

This indicates that restoration of myocardial function is a more complex process, which depends not only on the patency of coronary artery and the degree of myocardial lesions, but also on the geometric and hemodynamic forces acting at the level of different myocardial areas.^[11]^

Cardiac Magnetic Resonance Imaging (CMRI) provides reliable information on wall motion, perfusion, volume and mass of the left ventricle, considered nowadays the golden standard for assessment and quantification of myocardial scar.^[12–14]^ Assessment of myocardial viability is mandatory to evaluate the ability of ischemic tissue to recover after revascularization therapy. The gold standard for the myocardial viability evaluation is fluorodeoxyglucose-positron emission tomography.^[15–18]^ However, while CMRI examination and especially the late gadolinium enhancement sequences can easily visualize the transmural extent of the viable and non-viable myocardium in a simultaneously way, the nuclear scintigraphy can only indirectly assess the viable myocardium.^[18]^

At the same time, in non-infarct patients, functional impact of a coronary stenosis is crucial for selection of the appropriate therapy, since not all anatomically-significant coronary lesions produce myocardial ischemia, while other non-significant lesions proved to be associated with significant ischemia in the territory supplied by that coronary artery. The concept of subtended myocardial mass, reflecting the myocardial area suffering from the deficit of blood supply, has been introduced recently as a result of development of new machine learning algorithms associated with CCTA technology, which automatically quantify the subtended myocardial mass irrigated by a coronary lesion.^[19]^ This may replace the conventional ways to assess functional significance of a coronary lesion, mainly based on fractional flow reserve (FFR) calculation.

Evaluation of coronary circulation by CCTA and myocardial viability by CMRI, as well as assessment of coronary artery stenosis by CCTA and functional significance with invasive FFR, involve two different imaging techniques for one assessment, both of them expensive and time consuming, significantly increasing the cost of the patient care.

We describe the study protocol of the FUSE-HEART trial, a single-center, prospective, cohort study which aims to investigate the impact of a coronary artery stenosis on myocardial function and viability, based on advanced fusion imaging techniques derived from CCTA.^[20]^

### 1.2 Study objectives

The study primary objective is to investigate the impact of a coronary artery stenosis on myocardial function and viability, based on advanced fusion imaging techniques derived from CCTA.

The study secondary objective is to investigate the correlation between morphology and composition of atheromatous plaques located in a coronary artery and myocardial ischemia in the territory irrigated by the same coronary artery.^[20]^

## 2 Methods/design

### 2.1 Study design

This is a 2-year observational non-randomized clinical study, aiming to investigate the interrelation between the degree of coronary stenosis, plaque composition and myocardial ischemia. The period of 2 years consists of the 1-year follow-up period for major adverse cardiac events (MACE) events and the baseline screening.

### 2.2 Ethics

The FUSE-HEART protocol was authorized by the Ethics Committee for Scientific Research of the Cardio Med Medical Center (certificate of approval: 23/19.12.2017) and by the local Ethics Committee for Scientific Research of the University of Medicine and Pharmacy George Emil Palade of Tirgu-Mures, Romania (certificate of approval: 353 from 13 December 2017). All study procedures were verified in order to fulfil with the Declaration of Helsinki of 1975, and all subjects will assume a signed consent.

### 2.3 Study population

The study population will include 100 subjects with coronary lesions depicted by CCTA examination. The study population will consist in:

(1) patients with anatomically significant coronary lesions (at least 50% luminal narrowing) on native coronary arteries, or
(2) patients surviving an acute myocardial infarction, revascularized or not.

In all patients, presence of vulnerability features in the atheromatous plaques will be studied and the vulnerability score will be calculated for each plaque, consisting in one point added for each of the following vulnerability markers: positive remodeling, napkin-rink sign, presence of low-density plaque or spotty calcium within the plaque. In addition, following 3D fusion of the images of the coronary tree with the images reflecting wall motion, the correspondence between plaque morphology and composition on one hand, and wall motion in the corresponding distribution territory of that coronary artery, will be studied based on fused models.^[20]^

The study will be conducted over a period of 2 years. Patient assessment will be performed at baseline and at 1 year, and the projected recruitment period will be 1 year.

**Inclusion criteria:**

- Subjects undergoing CCTA examination, with at least one coronary artery lesion producing a luminal narrowing > 50%
- Conscious subjects in order to sign and assume a paper-based consent
- Subjects at the age of maturity

**Exclusion criteria:**

- Refusal or inability to sign the paper-based consent
- Allergy to iodine contrast agent used for CCTA acquisition
- Proved contraindications to CCTA evaluation
- Irregular or rapid heart rhythm
- Pregnancy or lactation
- Renal dysfunction (dialysis or serum levels of creatinine>1.5 mg/dL)
- Malignant disease in the last 5 years
- Life expectancy less than 2 years

### 2.4 Study settings

This clinical single center study will be runned in the Cardio Med Medical Center - Center of Advanced Research in Multimodality Cardiac Imaging, being funded by the Romanian Ministry of European Funds, the Romanian Government and the European Union, as part of the research grant number 103544 - Plaque IMAGE (contract number 26/01.09.2016), which was selected for funding following an international peer-review procedure within the national competition of research grants.^[20]^

### 2.5 Study groups

One hundred subjects meeting the selection criteria will be divided into 2 groups, thus, subjects with anatomically significant coronary lesions (at least 50% luminal narrowing) on native coronary arteries (group 1), and subjects surviving an acute myocardial infarction, revascularized or not (group 2).^[20]^

### 2.6 Study procedures and outcome assessment

The following data will be collected and recorded from all the subjects participating in the study: -baseline parameters of demography, medical history, cardiovascular risk factors, physical examination information, CCTA parameters, trans-thoracic echocardiography measurements, venous blood sample values, conventional 12-lead electrocardiography will be performed for all the subjects at baseline and follow-up.

#### 2.6.1 Biomarkers assays

At baseline, electrochemiluminescent immunoassay (IMMULITE 2000 XPi Immunoassay System, Siemens, United States of America), biochemistry, blood count and NT-proBNP will be determined. Also, inflammatory status will be determined and analyzed at the Advanced Medical and Pharmaceutical Research Center of the University of Medicine and Pharmacy Tirgu-Mures, Romania, based on serum high sensitive C-reactive protein levels determined using immunoturbidimetric assay (COBAS INTEGRA 400 plus analyzer, Roche Diagnostics, Switzerland).

MMP-9 and Il-6 levels will be also evaluated using ELISA method (Dynex DSX Automated ELISA System, Dynex Technologies; IMMULITE 2000 XPi Immunoassay System, Siemens, United States of America).^[20]^

#### 2.6.2 CCTA examination

All subjects will undergo a CCTA examination using a Siemens Somatom Definition 128 slices scanner (Siemens Healthcare GmbH, Erlangen, Germany). The acquired images will evaluate the coronary tree, especially the coronary stenoses, the presence of the plaques vulnerability markers such as: napkin ring sign, necrotic core, spotty calcifications, low density plaque, positive remodeling. Duke Jeopardy score will be calculated based on CCTA images.

#### 2.6.3 Cardiac Transthoracic Echocardiography assessment

2D Echocardiography will be performed at baseline using Vivid E9 echocardiography system (General Electric Vingmed Ultrasound, Horten, Norway) for assessment of myocardial contractility. Moreover, all cardiac diameters, volumes and ejection fraction will be evaluated.

#### 2.6.4 Fusion imaging protocol

Computational CT analysis will be performed using a Siemens SyngoVia software (Siemens Medical Solutions, Erlangen Germany), consisting in 3D reconstruction of the coronary arteries and myocardial motion.

**Figure 1:**
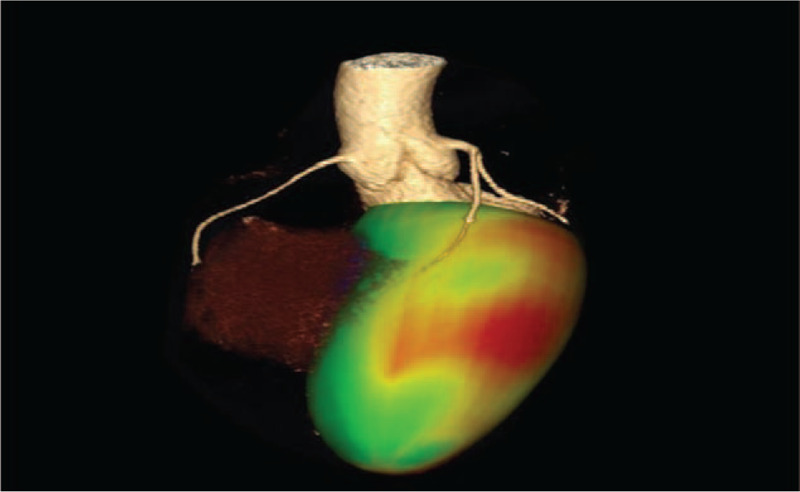
CCTA hybrid image which illustrates the coronary tree revealing a severe stenosis in the medial segment of LAD fussed with 3D motion map indicating hypokinesis in the territory of the stenotic artery. LAD = left anterior descending.

Coronary plaques advanced analysis will be performed semi-automatically using an image algorithm which detects the vascular contour, on the same research software using the imagistic platform dedicated to the coronary analysis (SyngoVia Frontier Coronary Plaque Analysis, Siemens, Erlangen, Germany), and the coronary arteries will be individually reconstructed around their axis. The 3D images of the coronary tree will be superimposed on the 3D images of myocardial tissue, color-coded in accordance with the contractility level of each myocardial area. Figure 1 represents an illustration of the first prototype obtained as a result of fusing imaging, fusing images of the coronary tree with images of the corresponding myocardial regions irrigated by each coronary artery.

### 2.7 Study time

The FUSE Heart study will be conducted in a period of two years, from January 2021 to December 2022.

### 2.8 Outcomes

The primary outcome of the study is to evaluate the rate of MACE events related to myocardial ischemia at 1-year post assessment, in correlation with the degree of coronary artery stenosis and myocardial ischemia or viability.^[20]^

The study secondary objective is to investigate the correlation between morphology and composition of atheromatous plaques located in a coronary artery and myocardial ischemia in the territory irrigated by the same coronary artery.^[20]^

### 2.9 Participation timeline

Baseline (day 0)

- Acquire and record a written consent from the subjects on customized paper file
- Confirm if the subjects meet the inclusion/exclusion criteria.
- Record demographic data, medication use history, tobacco and alcohol use history.
- Obtain data regarding physical examinations and 12-lead Electrocardiography recording.
- Collect blood samples (CBC, biochemistry, NT-proBNP, and inflammatory markers).
- CCTA acquisition, perform 2 D Echocardiography
- Image postprocessing – quantification of coronary stenosis, characterization of vulnerability markers, 3D reconstruction, extension and characterization of wall motion
- Deliver fused 3D images

Visit 1 (month 1,3,6,12)

- Obtain results of physical examinations, 12-lead electrocardiogram (ECG) and medical history.
- MACE assessment

Final study visit (month 12)

- Obtain results of physical examinations and medical history,12-lead ECG recording
- Perform transthoracic 2-Dimensional cardiac ultrasound
- End-point and MACE evaluation.^[20]^

**Study procedures:**

Medical history evaluation, clinical examination, laboratory tests assessment which consist of (complete blood count, biochemistry, inflammatory biomarkers: hs-CRP, MMP, interleukin 6, and N-terminal prohormone of brain natriuretic peptide)

12-lead ECG

2D transthoracic echocardiography

CCTA

Computerized postprocessing and fused images

**Figure 2:**
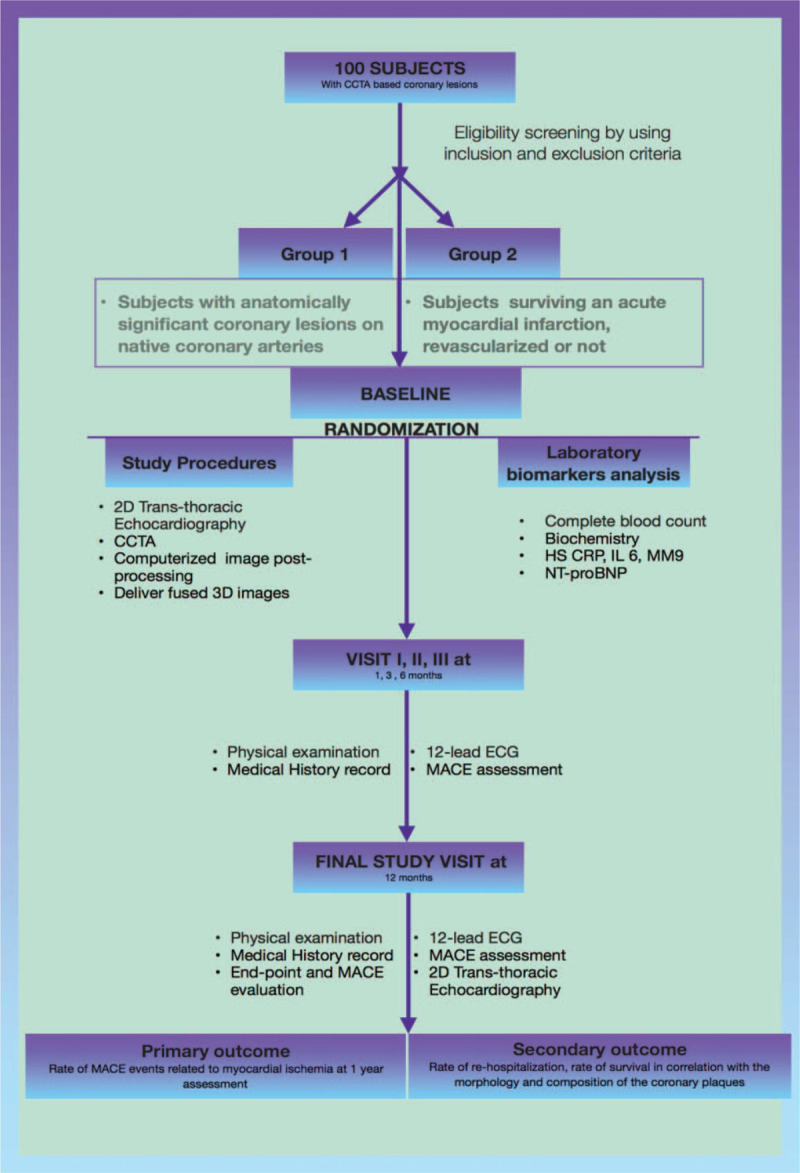
Illustrates the FUSE-HEART (FH) diagram with the flowchart that will be used in the study.

**Data collection**: All data will be recorded in a dedicated databank including medical history data, medication use history, imaging features provided by cardiac ultrasound, CCTA and fused images resulting from imaging post-processing.^[20]^ FUSE-HEART flowchart is presented in Figure 2.

### 2.10 Sample size

The trial sample size is represented by 100 subjects with anatomically significant coronary artery disease depicted by CCTA examination. The lot size estimation was done using the StatMate 2.0 software. A percent of 60% event-free study population was estimated for sample size calculation%, and a sample size of 50 subjects in each group is associated with a power of 90% for detection of an increase of 0.29 in MACE-free rates proportion, with a significance level (alpha) of 0.05 (two-tailed). Consequently, the study lot population including the 2 study groups of 50 patients each, was set at 100 patients.

### 2.11 Statistical analysis

The Graph Pad InStat 3.10 software will be used for statistical analysis, with a 5% level of significance. All data will be first checked for normality test. Continuous variables having normal distribution will be considered as mean ± standard deviation and will be compared using the t-test. Non-normally distributed data will be analyzed using the Mann-Whitney test. All the categorical variables, expressed in numbers and percentages, will be compared using the Fisher exact test.^[20]^

## 3 Discussion

This paper presents the protocol of a prospective, single-center clinical study which aims to determine the effectiveness of a new imaging tool based on fusion imaging in the complex assessment of coronary lesions. The present study was designed to validate a new and robust imaging method, superior to the conventional ones, used to determine the functional impact of coronary lesions on the myocardial tissue.

The impact of coronary artery stenosis on the myocardial contraction was addressed by multiple studies. It is well known that interventional assessment of coronary physiology during invasive coronary angiography may indicate the presence or absence of myocardial ischemia in the territory irrigated by the stenotic artery. Fractional flow reserve is an invasive procedure which may assess the impact of the coronary stenosis on the myocardial ischemia with an accuracy of 90%.^[21]^ The multicenter FAME study investigated the role of FFR-guided PCI compared to angiography-guided PCI, on clinical outcomes in patients undergoing coronary revascularization. After 5 years of follow up, MACE occurred more frequently in the angiographic-guided PCI, however with no statistical significance (31% vs 28%; RR-0.91, 95% CI, p-0.31).^[22]^ A recent analysis of the SCAAR registry (Swedish Angiography and Angioplasty Registry) that involved all patients treated with PCI in Sweden between 2005 to 2016, indicated that patients who underwent FFR-guided PCI had a 19% reduction in mortality after 5 years compared to those patients who performed angiography-guided PCI (*P* < .0001).^[23]^

However, these are invasive techniques that imply the risk associated with any interventional procedure. Therefore, identification of non-invasive imaging tools may help to better discriminate the lesions requiring treatment, which is particularly important especially in the case of multivessel disease. In a recent article, Savic et al found that multi-vessel disease remains an independent predictor for 6-year cardiovascular mortality (HR 1.55, 95% CI 1.11–2.06, *P* = .041), with a six-year cardiovascular mortality significantly higher than in patients with single-vessel disease (10.4% vs 4.6%, *P* < .001).^[24]^

Location of coronary lesions may also play a significant role on the clinical outcomes, since it has been demonstrated that an increased shear stress at the level of coronary bifurcations may accelerate the plaque vulnerabilisation process,^[25]^ while location in the proximal left anterior descendant artery is association with an increased risk of cardiac arrest.^[26]^

The FUSE-HEART study seeks to validate the usefulness and efficacy of fusion imaging by integrating anatomic information provided by CCTA with functional information provided by the same techniques, in order to identify the impact of a stenosis on subjacent myocardial contractility. In the case of multivessel disease, this fusion imaging may help to understand the complex interaction between different myocardial areas irrigated by stenotic arteries of the same heart.

At the same time, the impact of a coronary lesion results not only from its anatomical severity, but also from its composition. It has been demonstrated that vulnerable plaques triggering an acute cardiac event exhibit a different CCTA phenotype than plaques that remain un-ruptured. In a recent study by Licu et al, the most relevant vulnerability markers associated with an acute coronary event were presence of low attenuation within the plaque (*P* < .0001) and positive remodeling (*P* < .0001), while low attenuation plaques was the strongest independent predictor of acute myocardial infarction at 6 months (OR 8.18 [1.23–95.08], *P* = .04).^[27]^ Systemic inflammation may play also a significant role in the complex process of plaque vulnerabilisation and also in the evolution in the period of time post myocardial infarction^.^^[28,29]^ Therefore, the FUSE-Heart study will include a biomarker sub-study in order to investigate the association between systemic vulnerability and local factors that favor plaque vulnerabilisation, as well as the reflection of this association on plaque vulnerabilisation at a local level.

## 4 Conclusions

FUSE-HEART will be the first study based on fusion imaging that will investigate the impact of a coronary lesion on myocardial contractility, aiming to validate fusion-imaging as an effective alternative tool in the CCTA assessment of coronary circulation.

## Author contributions

**Conceptualization**: Alexandra Gorea Stanescu, Benedek Imre, Diana Opincariu, Mihaela Ratiu, Roxana Hodas, Theodora Benedek.

**Data curation**: Alexandra Gorea Stanescu, Diana Opincariu.

**Formal analysis**: Alexandra Gorea Stanescu, Theodora Benedek.

**Investigation**: Diana Opincariu, Alexandra Gorea Stanescu, Theodora Benedek.

**Methodology**: Imre Benedek, Theodora Benedek.

**Resources**: Imre Benedek.

**Supervision**: Imre Benedek, Theodora Benedek.

**Visualization**: Mihaela Ratiu.

**Writing – original draft**: Alexandra Gorea Stanescu, Benedek Imre, Diana Opincariu, Roxana Hodas, Benedek Theodora.
